# Cytomegalovirus as a potential trigger for acute coronary syndrome: A case of NSTEMI in a previously healthy patient

**DOI:** 10.21542/gcsp.2025.59

**Published:** 2025-12-31

**Authors:** Hussam Al Hennawi, Muhammad Salman Sabri, David Teng, Maxwell Berman, Botros Shenoda

**Affiliations:** 1 Jefferson Abington Hospital, Department of Internal Medicine, PA, USA; 2 Sidney Kimmel Medical College at Thomas Jefferson University, PA, USA

## Abstract

Cytomegalovirus (CMV) infection has been implicated in endothelial dysfunction and atherogenesis, yet its role as a potential trigger for acute coronary syndrome (ACS) remains underrecognized. We present a case of a 32-year-old male with no prior medical history who developed non-ST elevation myocardial infarction (NSTEMI) and was subsequently found to have active CMV infection. The patient had no known history cardiovascular disease, though subsequent testing did reveal additional underlying risk factors. This case highlights the need to consider infectious etiologies, including CMV, as contributors to ACS in young, otherwise apparently healthy individuals. Understanding the interplay between viral infections and atherosclerotic disease may lead to improved risk stratification and management of patients presenting with ACS.

## Introduction

Acute coronary syndrome (ACS) is traditionally associated with well-established risk factors such as hypertension, hyperlipidemia, diabetes, and smoking. However, there is increasing evidence that infectious agents, including cytomegalovirus (CMV), may contribute to endothelial dysfunction and plaque destabilization, potentially leading to acute myocardial infarction (MI) in patients with minimal conventional risk factors^1^. The herpesvirus family, which includes CMV, are some of the most ubiquitous of all human infections. In the general population, the global seroprevalence of CMV is estimated to be 83%^2^. CMV has been linked to atherogenesis through mechanisms such as chronic inflammation, endothelial injury, and immune-mediated plaque destabilization^3^. Despite these associations, CMV remains an underappreciated factor in ACS pathophysiology. Here, we present a case of a young, ostensibly healthy male with NSTEMI and active CMV infection, emphasizing the need for further investigation into infectious triggers of ACS.

### Case presentation

A 32-year-old male with no known significant past medical history presented to the emergency department with burning chest pain radiating to the left shoulder, pleuritic in nature, and associated with shortness of breath. Symptoms began one day prior to admission and were accompanied by a fever of 101°F. He denied recent illness or sick contacts.

On arrival, his vital signs were as follows: temperature 98°F, heart rate 117 bpm, blood pressure 132/81 mmHg, and respiratory rate 20 breaths per minute with normal oxygen saturation on room air. He appeared uncomfortable and diaphoretic, but his cardiovascular and pulmonary examinations were unremarkable. Social history was negative for tobacco or e-cigarette usage. Family history was not significant for premature coronary artery disease or sudden cardiac death in immediate relatives. However, it was noted that the patient’s maternal grandfather had an unspecified cardiac disease. There was no documented history of hyperlipidemia, hypertension, or diabetes. Given this information, further genetic testing for hereditary hyperlipidemias was not indicated at this time^4^.

Laboratory findings were significant for a total bilirubin of 1.3 mg/dL, aspartate aminotransferase (AST) of 171 IU/L, alanine aminotransferase (ALT) of 55 IU/L, high-sensitivity troponin T of 2027 ng/L, and a white blood cell count of 14.2 B/L. His hemoglobin A1c was 5.9%. The lipid panel showed total cholesterol of 187 mg/dL, LDL of 113 mg/dL, and an elevated lipoprotein (a) of 142.8 mg/dL. COVID-19 and influenza tests were negative, and blood cultures showed no growth.

**Figure 1.**
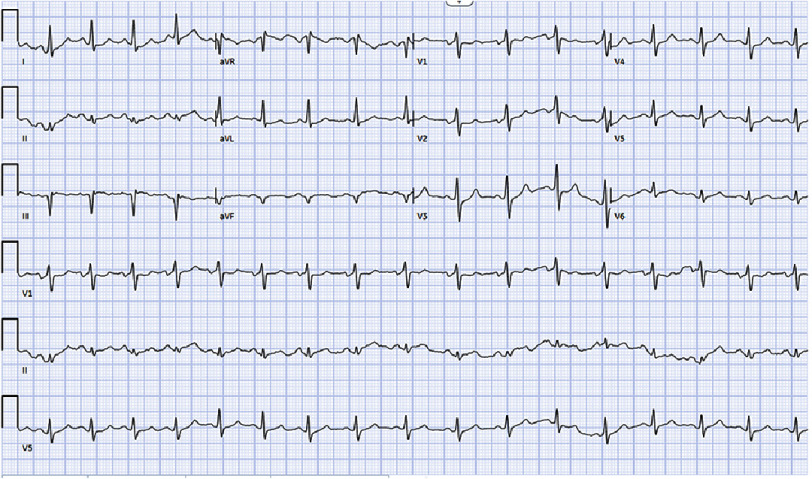
EKG demonstrated ST segment depressions in the inferior leads.

An electrocardiogram (EKG) demonstrated findings consistent with inferior infarction (Figure 1).

**Figure 2.**
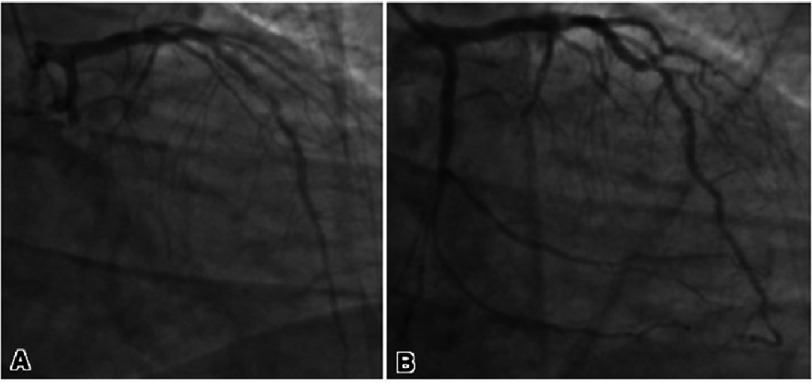
Pre-vascularization coronary angiography demonstrating acute thrombotic occlusion of the mid left circumflex artery, subtotal to total occlusion of proximal to mid right coronary artery with left to right collateral filling of the right posterior descending artery and severe focal mid LAD stenosis (A). Successful culprit lesion percutaneous intervention of the mid-left circumflex stenosis with post drug-eluting stent (B).

A transthoracic echocardiogram revealed a normal left ventricular ejection fraction without structural abnormalities. The patient was treated with aspirin and symptom management, but his chest pain persisted, and he remained diaphoretic. Coronary angiography revealed triple vessel disease (Figure 2).

**Figure 3.**
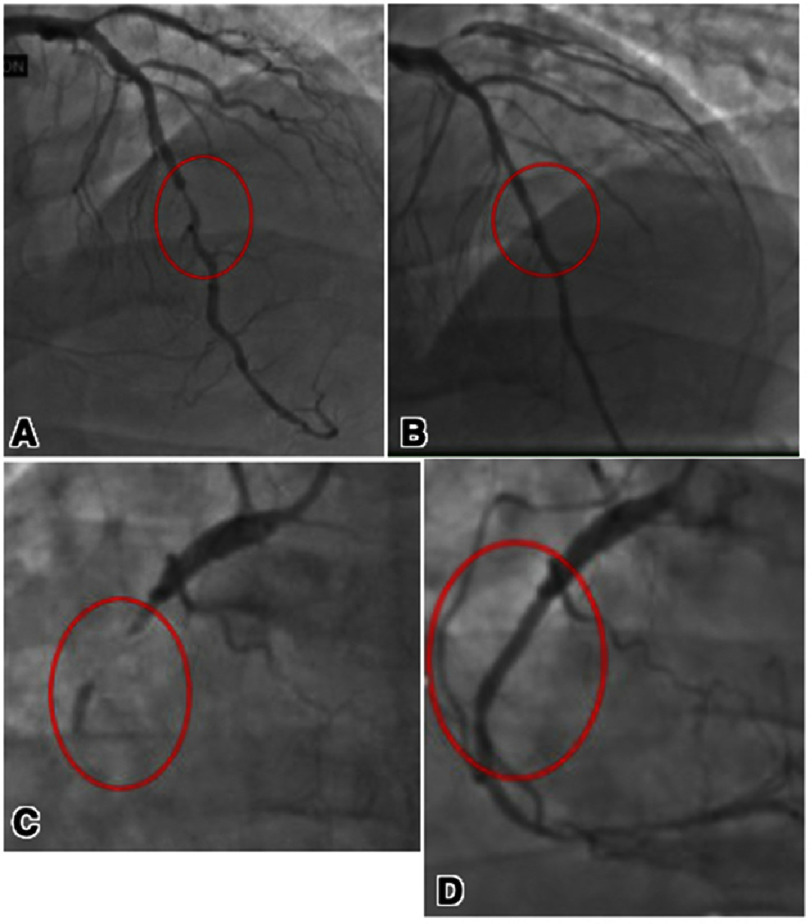
Repeat coronary angiography demonstrating widely patent stent in the left circumflex. Severe mid left anterior descending stenosis with successful drug-eluting stent (A, B). Chronic total occlusion of the proximal to mid right coronary artery with bridging collaterals post successful drug-eluting stent (C, D). The distal RCA after revascularization is of small caliber and has moderate diffuse stenosis.

Despite aggressive medical therapy, the patient continued to experience chest pain and became hemodynamically unstable the following day. He was taken for repeat catheterization, which revealed a patent left circumflex stent with persistent vessel disease. He underwent percutaneous intervention to the left anterior descending artery and right coronary artery (Figure 3) with subsequent symptom resolution. A post-catheterization TTE showed no notable cardiac dysfunction. The patient was subsequently discharged on dual antiplatelet therapy with aspirin and ticagrelor, along with high-dose statin therapy.

Two weeks following discharge from the hospital, the patient presented in the outpatient setting with concerns of a fever. Infectious and autoimmune etiologies were considered in the differential and screening tests were gathered. Laboratory values showed some inflammatory markers were elevated, notably an ESR of 81 mm/hr and a CRP 19 mg/L; no IL-6 testing performed. Laboratory testing for tuberculosis and hepatitis A, B, and C all returned negative. EBV testing revealed a positive EBNA-IgG, EBV VCA IgG, and EBV VCA IgM, which was consistent with recent infection. Serologic testing for CMV revealed an initial CMV IgG <0.60 U/mL and a positive IgM antibody titer of 57.4 AU/mL, suggestive of active infection. As the CMV IgG test was negative, CMV IgG avidity testing was not completed. A CMV PCR was not obtained at this time, and the patient was not tested for HSV, chlamydia, or mycoplasma.

The manifestations of illness were mild and resolving at the time of presentation; it was decided that antivirals were not necessary for treatment.

Another follow up visit was scheduled for 3 months later, where CMV testing was negative for IgG and IgM antibodies. Notably the patient’s symptoms had resolved, and no further studies were performed.

## Discussion

CMV has been increasingly recognized as a contributor to cardiovascular disease through mechanisms involving chronic inflammation, endothelial dysfunction, and immune-mediated vascular injury^5^. CMV infection can induce a prothrombotic state, promote arterial inflammation, and contribute to plaque instability, potentially precipitating acute coronary events.

Mechanistically, CMV is thought to contribute to ACS through several interrelated pathways. First, CMV can infect endothelial cells, leading to direct endothelial injury and increased expression of adhesion molecules such as intercellular adhesion molecule-1 (ICAM-1) and vascular cell adhesion molecule-1 (VCAM-1). This promotes leukocyte adhesion and migration, fostering a pro-inflammatory microenvironment conducive to atherogenesis.

Second, CMV infection induces a systemic inflammatory response, increasing levels of pro-inflammatory cytokines such as tumor necrosis factor-alpha (TNF-*α*), interleukin-6 (IL-6), and interferon-gamma (IFN-*γ*), which contribute to plaque destabilization. These inflammatory mediators can weaken the fibrous cap of atherosclerotic plaques, making them more prone to rupture, a key event in ACS pathogenesis^3^.

Furthermore, activated T lymphocytes stimulated by CMV antigens also play a role in plaque destabilization. CD8+ killer T lymphocytes and CMV-specific CD4+ T lymphocytes increase the production of ligands that bind receptors on endothelial cells releasing pro-inflammatory chemokines, such as fractalkine and IP-10. During primary infection with CMV, T cells that become differentiated following antigen presentation can be maintained during latency of the virus and a proinflammatory response of the immune system is activated^6^.

Additionally, CMV infection has been shown to upregulate procoagulant activity through increased expression of tissue factor and decreased fibrinolytic activity. This promotes a hypercoagulable state, increasing the risk of thrombus formation and subsequent coronary artery occlusion^7^.

Lastly, CMV has been implicated in direct smooth muscle cell proliferation within the arterial wall, leading to arterial remodeling and accelerated atherosclerosis. This process is mediated by CMV-encoded proteins that interfere with normal cellular signaling pathways, promoting atherosclerotic plaque progression^1^.

Owing to our patient presenting with late ACS and being immunocompetent, with improvement in symptoms, the decision was made to forgo treatment for underlying CMV infection. While mild elevations of liver enzyme and function testing suggested possible hepatic involvement, the patient did not have signs or symptoms of systemic CMV involvement of other organ systems such as encephalitis, retinitis, gastritis, colitis, etc.

This case underscores the need for heightened awareness of infectious triggers in ACS, particularly in young patients without previously known cardiovascular risk factors. It is important to acknowledge, however, the underlying cardiovascular risk factors in our patient revealed upon further workup—this includes the significant risk from notably elevated LDL and lipoprotein (a) levels, as well as the risk associated with pre-diabetic range hemoglobin A1c. This is further highlighted by the triple vessel disease seen on coronary angiography, suggesting a previously latent process of chronic atherosclerosis.

Other case reports have reported similar findings to our report, corroborating the impact CMV has on vascular or cardiovascular events. One case report of a young immunocompetent female showed that an acute CMV infection led to asymptomatic carotid intimal-medial thickening. The authors note that the patient had mild infectious mononucleosis-like symptoms such as fever and fatigue, but a bruit was noted on carotid artery exam leading to exploration of this finding. Following the resolution of her symptoms, it was noted on ultrasound and exam that there was now no evidence of carotid artery endothelial proliferation or other vascular changes^8^. Another case report looked at an immunocompetent patient with reactivation of CMV that had an acute MI. Although the patient had a complicated hospital course, it was shown that they were CMV positive and had signs of systemic infection including CMV colitis and hepatomegaly, showing an association of CMV infection with ACS^9^. Collectively, these two case reports suggest that CMV may precipitate acute vascular and cardiac events in young, immunocompetent patients, most likely through mechanisms such as direct endothelial injury, inflammation, and a prothrombotic state.

The clinical implications of our case may lead to the consideration of early testing for additional cardiovascular risk factors (such as blood pressure, diabetes, lipid profile –even if not overweight) in adults, even young adults, with positive serologies. There also should be consideration for possible early referral for cardiology evaluation.

An alternative explanation for this patient’s ACS could involve two contributing factors: Epstein-Barr virus (EBV) infection and elevated LDL levels. While CMV IgM was found to be positive on screening, this could be due to heterophile antibody cross reactivity leading to a false positive result. It is noted in the literature that false positive CMV IgM results may occur in patients infected with EBV or with immune disorders, as such CMV IgM testing should be interpreted with caution and in conjunction with other clinical and laboratory findings^10^. However, it should be noted that there is a paucity of guidelines for interpreting patients who are positive EBV and CMV IgM, which can make establishing a final diagnosis more complicated^11^.

Further research is necessary to determine whether screening for CMV and other viral pathogens in select populations may be beneficial in guiding preventive and therapeutic strategies. While it is noted that a large majority of the population is likely seropositive for CMV and it would be impractical to use this as an indication for screening for cardiovascular disease in the general population, select populations may benefit from targeted screening. Some studies show correlation between CMV seropositivity with mortality in cardiovascular disease, pointing to CMV as a potential independent risk factor for all-cause mortality^12^. Raising awareness of the cardiac implications of CMV infection, along with improving screening and diagnostic approaches, has the potential to significantly enhance patient outcomes.

## Conclusion

This case highlights the potential role of CMV infection as a trigger for ACS in an apparently healthy young adult. While traditional cardiovascular risk factors remain central to ACS pathophysiology, infectious agents such as CMV should also be considered in atypical presentations. Understanding the interplay between viral infections and coronary events may improve risk assessment and targeted interventions in at-risk populations.
